# The emergence of a novel sequence type of MDR *Acinetobacter baumannii* from the intensive care unit of an Egyptian tertiary care hospital

**DOI:** 10.1186/s12941-017-0208-y

**Published:** 2017-05-10

**Authors:** Doaa Mohammad Ghaith, Mai Mahmoud Zafer, Mohamed Hamed Al-Agamy, Essam J. Alyamani, Rayan Y. Booq, Omar Almoazzamy

**Affiliations:** 1 Department of Clinical and Chemical Pathology, Faculty of Medicine, Cairo University, Cairo, Egypt; 2 Department of Microbiology and Immunology, Faculty of Pharmacy, Ahram Canadian University, Giza, Egypt; 3 Department of Pharmaceutics, College of Pharmacy, King Saud University, PO box 2457, Riyadh, 11451 Saudi Arabia; 4 Department of Microbiology and Immunology, Faculty of Pharmacy, Al-Azhar University, Cairo, Egypt; 5 National Center for Biotechnology, King Abdulaziz City for Science and Technology, Riyadh, Saudi Arabia; 6 Department of Microbiology, Faculty of Science, Zagazig University, Zagazig, Egypt

**Keywords:** MDR**-***A. baumannii*, *bla*_OXA-23_-like, MLST

## Abstract

**Background and aim of work:**

*Acinetobacter baumannii* is known for nosocomial outbreaks worldwide. In this study, we aimed to investigate the antibiotic susceptibility patterns and the clonal relationship of *A. baumannii* isolates from the intensive care unit (ICU) of an Egyptian hospital.

**Methods:**

In the present study, 50 clinical isolates of multidrug resistant (MDR)-*A. baumannii* were obtained from patients admitted into the ICU from June to December 2015. All isolates were analyzed for antimicrobial susceptibilities. Multiplex PCR was performed to detect genes encoding oxacillinase genes (*bla*_OXA-51_-like, *bla*_OXA-23_-like, *bla*_OXA-24_-like, and *bla*_OXA-58_-like). Multilocus sequence typing (MLST) based on the seven-gene scheme (*gltA, gyrB, gdhB, recA, cpn60, gpi, rpoD*) was used to examine these isolates.

**Results:**

All *A. baumannii* clinical isolates showed the same resistance pattern, characterized by resistance to most common antibiotics including imipenem (MIC ≥ 8μ/mL), with the only exception being colistin. Most isolates were positive for *bla*_OXA-51_-like and *bla*_OXA-23_-like (100 and 96%, respectively); however, *bla*_OXA-24_-like and *bla*_OXA-58_-like were not detected. MLST analysis identified different sequence types (ST195, ST208, ST231, ST441, ST499, and ST723) and a new sequence type (ST13929) with other sporadic strains.

**Conclusions:**

MDR *A. baumannii* strains harboring *bla*_OXA-23_-like genes were widely circulating in this ICU. MLST was a powerful tool for identifying and epidemiologically typing our strains. Strict infection control measures must be implemented to contain the worldwide spread of MDR *A. baumannii* in ICUs.

## Background

The clinical care of intensive care unit (ICU) patients with infections has been complicated by the emergence and spread of extremely drug-resistant (XDR) *Acinetobacter baumannii* strains [1]. Due to scarce current therapeutic options; higher infection rates; poor patient outcomes because of life-threatening infections, including ventilator-related pneumonia, sepsis, urinary tract infections, and skin and soft tissue disorders may occur [1, 2].

Extensively drug-resistant (XDR) *A. baumannii* strains exhibiting resistance to three or more antibiotic classes, except for polymyxins, have been recently described in nosocomial outbreaks [2, 3]. The essential role of *A. baumannii* resistance to carbapenems, is mediated by oxacillinases (OXA-class D) and, less frequently, by metallo-β-lactamases (MBL-class B) [4, 5]. The class D carbapenemases are the most predominant carbapenemases in *A. baumannii*. They are categorized into six subclasses: intrinsic chromosomal OXA-51-like, the acquired OXA-23-like, OXA-24/40-like, OXA-58-like, OXA-143-like, and OXA-235-like β-lactamases [6]. In this study, we aimed to investigate the antimicrobial susceptibility, class D carbapenemases and clonal relationship of *A. baumannii* strains isolated from a tertiary care hospital ICU in Egypt.

## Methods

The study was carried out in EL Sheikh Zayed hospital which provides tertiary care from specialists and consultants after referral (in orthopedic, trauma, neuro/spine surgeries) from primary care and secondary care hospitals in Egypt. A lab-based surveillance was performed over a period of 6 months (June–December 2015) after the isolation of five MDR *A. baumannii* strains in a period of 1 week showing the same phenotypic characteristics.

### Bacterial strains

All clinical samples of the patients admitted during the above-mentioned period were processed at the microbiology unit. All samples were cultured on blood agar and MacConkey agar (Oxoid Co. England). All culture plates were incubated aerobically at 35 °C for 24–48 h. Identification of isolated organisms was performed by conventional biochemical reactions. During the experimental period, 50 *A. baumannii* non-duplicate strains were isolated.

### Antimicrobial susceptibility testing

Susceptibility testing was performed by the disc diffusion method (Modified Kirby-Bauer technique) using Mueller–Hinton agar and aerobic incubation at 35 °C for 16–18 h. Antimicrobial discs containing imipenem (10 μg), meropenem (10 μg), gentamicin (10 μg), ciprofloxacin (5 μg), amikacin (30 μg), cotrimoxazole (25 μg), cefepime (30 μg), cefotaxime (30 μg), cefotaxime/clavulanic acid (30/10 μg), aztreonam (30 μg), ceftazidime (30 μg), ceftazidime/clavulanic acid (30/10 μg), amoxicillin/clavulanic acid (20/10 μg), and cefoxitin (30 μg) were obtained from Oxoid Co. (Oxoid Limited, Basingstoke, Hampshire, England) [7].

Multidrug resistance was defined in this analysis as resistance to three or more representatives of the following classes of antibiotics: fluoroquinolones, extended-spectrum cephalosporins, aminoglycosides, and carbapenems [8].

*Escherichia coli* ATCC 25922, *Pseudomonas aeruginosa* ATCC 27853, and *Staphylococcus aureus* ATCC 29213 were used as reference strains for susceptibility testing per Clinical and Laboratory Standards Institute (CLSI, 2015) guidelines and interpretations [7].

Minimum inhibitory concentrations (MICs) were determined by broth microdilution and interpreted using CLSI, 2015 guidelines [7].

The presence of *A. baumannii* genes encoding oxacillinases (*bla*_OXA-23_-like, *bla*_OXA-24_-like, *bla*_OXA-51_-like, and *bla*_OXA-58_-like) was assessed in all 50 isolates using multiplex PCR.

### Multiplex PCR assay

The sequences of *bla*_OXA_ alleles encoding carbapenemases were aligned and group-specific regions were identified using BioEdit software (http://www.mbio.ncsu.edu/BioEdit/bioedit.html). The primers: 5′-TAA TGC TTT GATCGG CCT TG and 5′-TGG ATT GCA CTT CAT CTT GG were used to amplify a 353 bp fragment of genes encoding the intrinsic OXA-51-like enzymes of *A. baumannii* [9].

A set of primers were designed to amplify OXA-23-like genes (501 bp: 5′-GAT CGG ATT GGA GAA CCAGA and 5′-ATT TCT GAC CGC ATT TCC AT), OXA-24-like genes (246 bp: 5′-GGT TAG TTG GCC CCC TTA AA and 5′-AGT TGA GCG AAA AGG GGA TT), and OXA-58-like genes (599 bp: 5′-AAG TAT TGG GGC TTG TGC TG and 5′-CCCCTCTGCGCTCTACATAC) [9]. The primers were evaluated separately against control strains and then in a multiplex format. The amplification conditions were: initial denaturation at 94 °C for 5 min, 30 cycles of 94 °C for 25 s, 52 °C for 40 s, and 72 °C for 50 s, and a final elongation at 72 °C for 6 min [9].

### Multilocus sequence typing

MLST analysis was performed per the protocol of the Pasteur Institute. Fragments of seven internal housekeeping genes (*gltA, gyrB, gdhB, recA, cpn60, gpi,* and *rpoD*) were amplified and sequenced as previously described [10]. Briefly, PCR amplifications were performed with a MasterCycler Nexus (Eppendorf, Hamburg, Germany) with an initial denaturation at 94 °C for 5 min, followed by 35 cycles of denaturation at 94 °C for 1 min, annealing at 55 °C for 1 min, and extension at 72 °C for 2 min, and a 4-min final extension at 72 °C. The amplicons were verified by agarose gel electrophoresis and were subsequently purified for bidirectional Sanger sequencing reactions. Multiple allele sequences were assigned for each locus with an arbitrary allele number to obtain characterization of sequence types (STs) for each *A. baumannii* isolate. Each sequence was compared with sequences deposited in the Institute of Pasteur MLST schema (http://pubmlst.org/perl/bigsdb/bigsdb.pl?db=pubmlst_abaumannii_pasteur_seqdef).

## Results

**Fig. 1:**
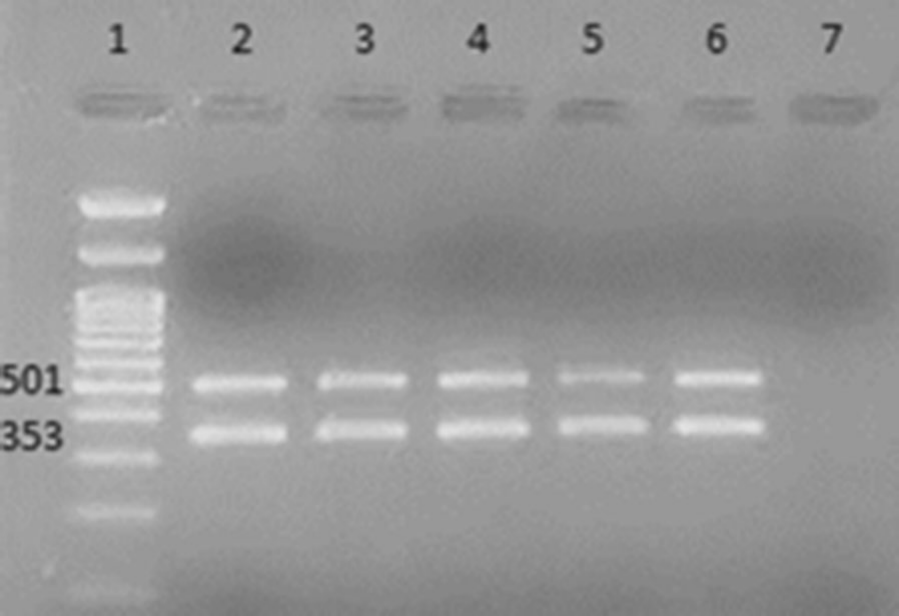
Results of multiplex PCR for detection of *bla*_OXA-51_-like, *bla*_OXA-23_-like, *bla*_OXA-24_-like, and *bla*_OXA-58_-like genes. *Lane 1* 100 bp DNA Ladder, *Lane 2* positive control, *Lanes 3*–*6 A. baumannii* clinical isolates showing *bla*_OXA-51_-like and *bla*_OXA-23_-like positivity (353 and 501 bp, respectively). *bla*_OXA-24_-like and *bla*_OXA-58_-like were not detected at (246 and 599 bp) respectively. *Lane 7* negative control

**Fig. 2:**
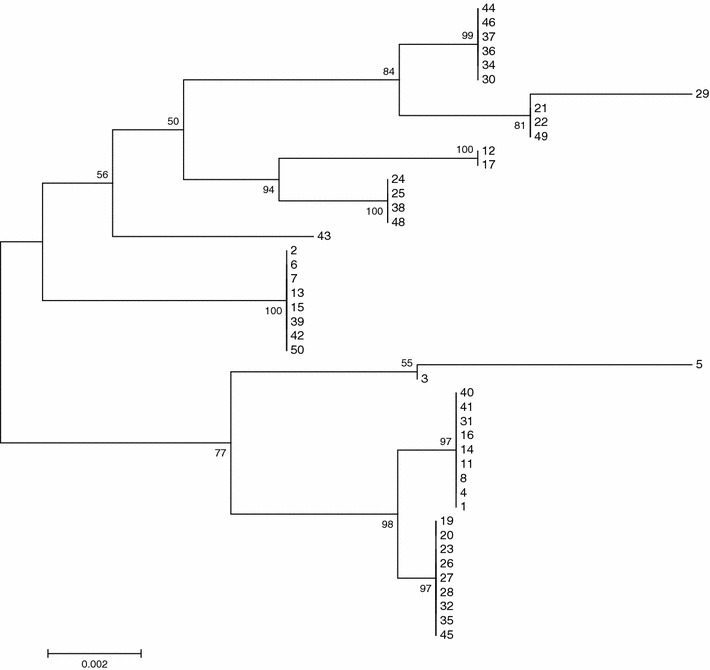
Phylogenetic tree of 50 *A. baumannii* strains built based on the maximum likelihood algorithm in the MEGA6 software [11] and the concatenated alleles of seven housekeeping genes with bootstrap values. The phylogenetic analysis identified ST195, ST208, ST231, ST441, ST499, and ST723 and the new sequence type ST13929. The numbers in the branches depict the sample ID of *A. baumannii*

Out of 358 patients admitted to ICU from June to December 2015, 56 (15.6%) patients were diagnosed with various types of hospital-acquired infections (HAI) as shown in Table 1. A total of 50 non-duplicate *A. baumannii* strains were isolated from different patient samples. Phenotypic antibiotic susceptibility testing for all *A. baumannii* isolates showed the same drug resistance pattern, characterized by resistance to all antibiotics used including imipenem, except for colistin. Genotypic analysis of *bla*_OXA-51_-like, *bla*_OXA-23_-like, *bla*_OXA-24_-like, and *bla*_OXA-58_-like genes by multiplex PCR (Fig. 1) showed that *bla*_OXA-51_-like and *bla*_OXA-23_-like were the most prevalent genes with 100 and 96% prevalence, respectively. However, *bla*_OXA-24_-like and *bla*_OXA-58_-like were not detected in the current study. Multilocus sequence typing (MLST) of the 50 clinical isolates of *A. baumannii* has yielded different sequence types; ST195, ST208, ST231, ST441, ST499, and ST723. Interestingly, a new sequence type ST13929 was identified among *A. baumannii* clinical isolates as shown in Table 2 and Fig. 2. *A. baumannii* ST13929 has been isolated from a young male (21 years old) patient who had no history of overseas travel. He was admitted to ICU at El Sheikh Zayed Specialized Hospital, Giza, Egypt on 5th of December 2014. The patient had multiple traumas due to motor car accident. After 7 days of ventilation the patient diagnosed to have ventilator associated pneumonia (VAP). Empirical antibiotic therapy of intravenous ceftriaxone/cefotaxime had been initiated. Endotracheal aspiration has been cultured on blood, chocolate and MacConkey agars. The recovered colonies had been identified as *A. baumannii.* Further genotypic identification was done by restriction analysis of 16S–23S rRNA spacer sequences using *Alu*I and *Nde*II. The isolate exhibited XDR towards imipenem (MIC > 32 mg/L), meropenem (MIC > 32 mg/L), ceftazidime (>256 mg/L), cefepime (MICs > 256 mg/L), gentamicin (MICs > 256 mg/L), amikacin (MICs > 256 mg/L), and ciprofloxacin (MICs > 32 mg/L). Tigecycline susceptibility was observed at MIC of 1 mg/L. The antimicrobial therapy was changed to tigecycline on day 11. The patient had spent 107 days in the hospital. The patient was alive after the hospitalization period. There was an ongoing XDR- *A. baumannii* outbreak in the institution in the same period and multiple isolates had been investigated.

**Table 1:** Patient’s data

	Age (mean ± SD)	Sex %		APACHE %		Length of stay (mean ± SD)	More than one device inserted %
		Male	Female	<15%	>15%		
VAP	(42.62 ± 17.63)	72%	28%	40%	60%	(48.96 ± 77.3)	(100%)
CLABSI	(41 ± 23.4)	88.8%	11.1%	33.3%	66.6%	(22.33 ± 11.4)	(55.55%)
CAUTI	(44.53 ± 22.1)	76.9%	23.0%	38.4%	61.5%	(82.307 ± 67.8)	(100%)
P value	0.900	0.535		0.880		0.131	0.00

*VAP* ventilator associated pneumonia, *CLABSI* central line associated blood stream infection, *CAUTI* catheter associated urinary tract infection, *APACHI* acute physiology and chronic health evaluation

**Table 2:** Sequence types (STs), allele profiles of 50 carbapenem-resistant *A. baumannii* isolates, carbapenem-hydrolyzing class D β-lactamase genes, minimum inhibitory concentration (MIC), site of isolation and patient outcome

	Sample ID	Site of isolation	Allele profile							ST	Carbapenem-hydrolyzing class D β-lactamase genes				MIC R ≥ 8 (mg/L)	Patient outcome
			*gltA*	*gyrB*	*gdhB*	*recA*	*cpn60*	*gpi*	*rpoD*		*bla*_OXA-51_	*bla*_OXA-23_	*bla*_OXA-24_	*bla*_OXA-58_	IMI	
*A. baumannii*	21	ETA	1	3	3	2	2	96	3	195	+	+	−	−	25	Deceased
*A. baumannii*	22	ETA	1	3	3	2	2	96	3	195	+	+	–	–	20	Deceased
*A. baumannii*	30	ETA	1	3	3	2	2	97	3	208	+	+	–	–	25	Deceased
*A. baumannii*	33	ETA	1	3	No gene	2	2	97	3	NA	+	+	–	–	25	Deceased
*A. baumannii*	34	ETA	1	3	3	2	2	97	3	208	+	–	–	–	35	Deceased
*A. baumannii*	36	ETA	1	3	3	2	2	97	3	208	+	+	–	–	20	Deceased
*A. baumannii*	37	ETA	1	3	3	2	2	97	3	208	+	+	–	–	30	Deceased
*A. baumannii*	44	Blood	1	3	3	2	2	97	3	208	+	+	–	–	25	Deceased
*A. baumannii*	46	Urine	1	3	3	2	2	97	3	208	+	+	–	–	25	Deceased
*A. baumannii*	47	Urine	1	3	No gene	2	2	97	3	NA	+	+	–	–	30	Discharged
*A. baumannii*	49	Urine	1	3	3	2	2	96	3	195	+	+	–	–	25	Deceased
*A. baumannii*	5	ETA	1	12	3	2	2	79	3	1114	+	+	–	–	20	Deceased
*A. baumannii*	29	ETA	1	87	3	2	2	96	3	13929^a^	+	+	–	–	20	Discharged
*A. baumannii*	24	ETA	1	107	12	10	23	195	26	723	+	+	–	–	25	Deceased
*A. baumannii*	25	ETA	1	107	12	10	23	195	26	723	+	+	–	–	25	Deceased
*A. baumannii*	38	ETA	1	107	12	10	23	195	26	723	+	+	–	–	30	Deceased
*A. baumannii*	48	urine	1	107	12	10	23	195	26	723	+	+	–	–	25	Deceased
*A. baumannii*	1	ETA	10	12	4	11	4	100	5	441	+	+	–	–	30	Deceased
*A. baumannii*	3	ETA	10	12	4	11	4	79	5	945	+	+	–	–	15	Deceased
*A. baumannii*	4	ETA	10	12	4	11	4	100	5	441	+	+	–	–	35	Discharged
*A. baumannii*	8	ETA	10	12	4	11	4	100	5	441	+	+	–	–	35	Deceased
*A. baumannii*	11	ETA	10	12	4	11	4	100	5	441	+	+	–	–	20	Deceased
*A. baumannii*	14	ETA	10	12	4	11	4	100	5	441	+	+	–	–	20	Deceased
*A. baumannii*	16	ETA	10	12	4	11	4	100	5	441	+	+	–	–	25	Discharged
*A. baumanii*	18	ETA	10	12	No gene	11	4	98	5	NA	+	+	–	–	20	Deceased
*A. baumanii*	19	ETA	10	12	4	11	4	98	5	231	+	+	–	–	20	Deceased
*A. baumannii*	20	ETA	10	12	4	11	4	98	5	231	+	+	–	–	20	Deceased
*A. baumannii*	23	ETA	10	12	4	11	4	98	5	231	+	+	–	–	15	Deceased
*A. baumannii*	26	ETA	10	12	4	11	4	98	5	231	+	+	–	–	25	Deceased
*A. baumannii*	27	ETA	10	12	4	11	4	98	5	231	+	+	–	–	20	Deceased
*A. baumannii*	28	ETA	10	12	4	11	4	98	5	231	+	+	–	–	20	Deceased
*A. baumannii*	31	ETA	10	12	4	11	4	100	5	441	+	+	–	–	25	Deceased
*A. baumannii*	32	ETA	10	12	4	11	4	98	5	231	+	+	–	–	20	Deceased
*A. baumannii*	35	ETA	10	12	4	11	4	98	5	231	+	+	–	–	25	Deceased
*A. baumannii*	40	ETA	10	12	4	11	4	100	5	441	+	+	–	–	25	Deceased
*A. baumannii*	41	Blood	10	12	4	11	4	100	5	441	+	+	–	–	25	Discharged
*A. baumannii*	45	ETA	10	12	4	11	4	98	5	231	+	+	–	–	20	Deceased
*A. baumannii*	12	ETA	12	17	12	1	29	102	39	236	+	+	–	–	35	Deceased
*A. baumannii*	17	ETA	12	17	12	1	29	102	39	236	+	+	–	–	35	Deceased
*A. baumannii*	2	ETA	24	92	96	11	49	162	26	499	+	+	–	–	20	Discharged
*A. baumannii*	6	ETA	24	92	96	11	49	162	26	499	+	+	–	–	25	Deceased
*A. baumannii*	7	ETA	24	92	96	11	49	162	26	499	+	+	–	–	20	Deceased
*A. baumannii*	13	ETA	24	92	96	11	49	162	26	499	+	+	–	–	15	Deceased
*A. baumannii*	15	ETA	24	92	96	11	49	162	26	499	+	+	–	–	15	Deceased
*A. baumannii*	39	ETA	24	92	96	11	49	162	26	499	+	+	–	–	35	Deceased
*A. baumannii*	42	Blood	24	92	96	11	49	162	26	499	+	+	–	–	25	Deceased
*A. baumannii*	50	Swab	24	92	96	11	49	162	26	499	+	+	–	–	20	Deceased
*A. baumannii*	43	Blood	28	38	45	1	16	66	2	1089	+	–	–	–	25	Deceased
*A. baumannii*	9	ETA	44	73	No gene	11	44	‡	4	NA	+	+	–	–	40	Deceased
*A. baumannii*	10	ETA	44	73	No gene	11	44	‡	4	NA	+	+	–	–	20	Discharged

*ETA* endotracheal aspirate, + positive, − negative

^a^ New sequence type (ST13929)

^‡^ *gpi* 173, 1 difference found. 33T → 33C

## Discussion

MDR *A. baumannii* is a problematic, multidrug-resistant pathogen identified in healthcare settings worldwide, especially in ICUs [12]. *A. baumannii* has a notable ability to capture and express resistance genes. All resistance mechanisms including target modification, efflux pump expression, and enzymatic inactivation have been described in *A. baumannii* [13].

In the current study, five MDR *A. baumannii* strains were isolated over 1 week from the same ICU. All isolates showed the same phenotypic characteristics which prompted us to start a survey study of the antimicrobial susceptibility and clonal relationship of *A. baumannii* strains isolated from this ICU.

All our isolates were resistant to imipenem. The main role of the *A. baumannii* resistance to carbapenems is mediated by oxacillinases and, less frequently, by metallo-β-lactamases [4, 5].

*bla*_OXA-23_-like, *bla*_OXA-24/40_-like, and *bla*_OXA-58_-like genes have been repetitively reported in *A. baumannii* outbreaks from diverse parts of the world. The localization of numerous β-lactamase genes on plasmids facilitates their horizontal mobilization from one bacterium to another [13, 14].

All our isolates harbored the *bla*_OXA-51_-like gene, which is ubiquitous in *A. baumannii* [15]. *bla*_OXA-23_ was the most universally identified gene, while *bla*_OXA-24_-like and *bla*_OXA-58_-like genes were not detected in any strain. *bla*_OXA-23_ is the most prevalent carbapenemase-encoding gene in the Mediterranean region. This might be explained by the higher carbapenemase activity of *bla*_OXA-23_ and/or acquisition of carbapenem resistance through horizontal gene transfer [16–18]. The *bla*_OXA-23_ gene was either encoded on the chromosome or on plasmids and was associated with four dissimilar genetic structures, with the most common being transposons Tn2006. *bla*_OXA-23_ has been reported in different regions of the Middle East, The United Arab Emirates, Algeria, Libya, Bahrain, and recently, Qatar [16, 19].

Mugnier et al. found an isolate from Egypt harboring plasmid containing *bla*_OXA-23_. This finding might indicate the prevalence of the genetic environment of *bla*_OXA-23_ in Egyptian isolates [16]. Moreover, a recent study including three Egyptian hospitals revealed the emergence and spread of *bla*_NDM-1_ and *bla*_OXA-23_ in addition to the co-occurrence of 16S rRNA methylase *armA* with *bla*_NDM-1_ and *bla*_OXA-23_ in 27 distinct sequence types, 11 of which were novel among *A. baumannii* clinical isolates [20].

Per MLST results, ST195, ST208, ST231, ST441, ST499, and ST723 were the most prevalent isolates. Another Egyptian study illustrated the large diversity found within the strains where ten distinct sequence types (STs) were identified, ST408–ST414, ST331, ST108, and ST208 [21]. However, a study showed that the most prevalent sequence types in the gulf area were ST195, ST208, ST229, ST436, ST450, ST452, and ST499 [22].

Taking into consideration that ST208 is the ancestor strain of several STs including ST89, ST88, ST190, ST225, and ST75, it has been identified in different parts of the world such as Japan, China, Thailand, Korea, Italy, Australia, Portugal, and the Czech Republic [23].

In conclusion, MDR *A. baumannii* strains harboring the *bla*_OXA 23_-like gene were widely circulating in our ICU. MLST provided us with a powerful tool for identifying and epidemiologically typing our strains. Studying the epidemiology of HAIs is urgent to prevent the clonal dissemination of antibiotic-resistant pathogens, not only in hospital settings, but in the community, as well. Strict infection control measures and antimicrobial stewardship programs are necessary to contain the worldwide spread of MDR *A. baumannii*. Proving the clonal relation between clinical isolates emphasizes the importance of surveillance programs and strict IC measures that would influence decision-making and health policy.

## References

[1] Ghaith DM, Hassan RM, Hasanin AM (2015). Rapid identification of nosocomial *A. baumannii* isolated from a surgical intensive care unit in Egypt. Ann Saudi Med.

[2] Hasanin A, Mukhtar A, El-Adawy A, Elazizi H, Lotfyn A, Nassar H, Ghaith D (2016). Ventilator associated pneumonia caused by extensive-drug resistant *Acinetobacter* species: colistin is the remaining choice. Egypt J Anaesth..

[3] Helal S, El Anany M, Ghaith D, Rabeea S (2015). The role of MDR- *A. baumannii* in orthopedic surgical site infections. Surg Infect.

[4] Poirel L, Nordmann P (2006). Carbapenem resistance in *A. baumannii*: mechanisms and epidemiology. Clin Microbiol Infect.

[5] Higgins PG, Dammhayn C, Hackel M, Seifert H (2010). Global spread of carbapenem resistant *A. baumannii*. J Antimicrob Chemother.

[6] Turton JF, Woodford N, Glover J, Yarde S, Kaufmann ME, Pitt TL (2006). Identification of *A. baumannii* by detection of the bla_OXA-51_-like carbapenemase gene intrinsic to this species. J Clin Microbiol.

[7] Clinical and Laboratory Standards Institute. Performance standards for antimicrobial susceptibility testing; twenty-fifth informational supplement. Document M100-S25. Wayne: CLSI; 2015.

[8] Magiorakos AP, Srinivasan A, Carey RB, Carmeli Y, Falagas ME, Giske CG (2012). Multidrug-resistant, extensively drug-resistant and pandrug-resistant bacteria: an international expert proposal for interim standard definitions for acquired resistance. Clin Microbiol Infect.

[9] Woodford N, Ellington M, Coelho J, Turton J, Ward M, Brown S, Amyes S, Livermore D (2006). Multiplex PCR for genes encoding prevalent OXA Carbapenemases in *Acinetobacter* spp.. Int J Antimicrob Agents.

[10] Alyamani EJ, Khiyami MA, Booq RY, Alnafjan BM, Altammami MA, Bahwerth FS (2015). Molecular characterization of extended-spectrum beta-lactamases (ESBLs) produced by clinical isolates of *Acinetobacter baumannii* in Saudi Arabia. Ann Clin Microbiol Antimicrob..

[11] Tamura K, Stecher G, Peterson D, Filipski A, Kumar S (2013). MEGA6: molecular evolutionary genetics analysis version 6.0. Mol Biol Evol.

[12] Hasanin A, Eladawy A, Mohamed H, Salah Y, Lotfy A, Mostafa H, Ghaith D, Mukhtar A (2014). Prevalence of extensively drug-resistant gram negative bacilli in surgical intensive care in Egypt. Pan Afr Med J..

[13] Higgins PG, Dammhayn C, Hackel M, Seifert H (2010). Global spread of carbapenem-resistant *A. baumannii*. Br Soc Antimicrob Chemother..

[14] Djahmi N, Dunyach-Remy C, Pantel A, Dekhil M, Sotto A, Lavigne JP (2014). Epidemiology of carbapenemase-producing *Enterobacteriaceae* and *A. baumannii* in Mediterranean countries. Biomed Res Int..

[15] Hamouda A, Evans BA, Towner KJ (2010). Amyes SBG. Characterization of epidemiologically unrelated *A. baumannii* isolates from four continents by use of multilocus sequence typing, pulsed-field gel electrophoresis, and sequence-based typing of blaOXA-51-like genes. J Clin Microbiol.

[16] Mugnier PD, Poirel L, Naas T, Nordmann P (2009). Worldwide dissemination of the blaOXA-23 carbapenemase gene of *Acinetobacter Baumannii*. Emerg Infect Dis.

[17] Minandri F, D’Arezzo S, Antunes LCS, Pourcel C, Principe L, Petrosillo N, Visca P (2011). Evidence of diversity among epidemiologically related carbapenemase-producing *Acinetobacter Baumannii* strains belonging to international clonal lineage II. J Clin Microbiol.

[18] Grosso F, Quinteira S, Peixe L (2011). Understanding the dynamics of imipenem-resistant *A. baumannii* lineages within Portugal. Clin Microbiol Infect Dis..

[19] Rolain JM, Loucif L, Al-Maslamani M, Elmagboul E, Al-Ansari N, Taj-Aldeen S (2016). Emergence of multidrug-resistant *A. baumannii* producing OXA-23 carbapenemase in qatar. New Microbes New Infect..

[20] El-Sayed MAEG, Amin MA, Tawakol WM, Loucif L, Bakour S, Rolain JM (2015). High prevalence of blaNDM-1 carbapenemase-encoding gene and 16S rRNA *armA* methyltransferase gene among *Acinetobacter baumannii* clinical isolates in Egypt. Antimicrob Agents Chemother.

[21] Al-Hassan L, El Mehallawy H, Amyes SG (2013). Diversity in *Acinetobacter baumannii* isolates from paediatric cancer patients in Egypt. Clin Microbiol Infect.

[22] Zowawi HM, Sartor AL, Sidjabat HE, Balkhy HH, Walsh TR, Al Johani SM (2015). Molecular epidemiology of carbapenem-resistant *Acinetobacter baumannii* isolates in the Gulf Cooperation Council states: dominance of OXA-23-type producers. J Clin Microbiol.

[23] Liu F, Zhu Y, Yi Y, Lu N, Zhu B, Hu Y (2014). Comparative genomic analysis of *Acinetobacter baumannii* clinical isolates reveals extensive genomic variation and diverse antibiotic resistance determinants. BMC Genomics..

